# Resistance Profile, Terbinafine Resistance Screening and MALDI-TOF MS Identification of the Emerging Pathogen *Trichophyton indotineae*

**DOI:** 10.1007/s11046-024-00835-4

**Published:** 2024-03-14

**Authors:** Roelke De Paepe, Anne-Cécile Normand, Silke Uhrlaß, Pietro Nenoff, Renaud Piarroux, Ann Packeu

**Affiliations:** 1 Mycology and Aerobiology Department, Sciensano, 1050 Brussels, Belgium; 2 Parasitology/Mycology Department, AP-HP, Hôpitaux de Paris, 75013 Paris, France; 3 Labopart Medical Laboratories Leipzig-Mölbis, 04571 Rötha OT Mölbis, Germany; 4 BCCM/IHEM Fungal Collection, Mycology and Aerobiology Section, Sciensano, 1050 Brussels, Belgium

**Keywords:** *Trichophyton indotineae*, Medical mycology, Antifungal susceptibility testing, MALDI-TOF MS, Terbinafine, Naftifine, Amorolfine, Dermatophytosis

## Abstract

The emerging pathogen *Trichophyton indotineae*, often resistant to terbinafine (TRB), is known to cause severe dermatophytoses such as *tinea corporis* and *tinea cruris*. In order to achieve successful treatment for these infections, insight in the resistance profile of *T. indotineae* strains and rapid, reliable identification is necessary. In this research, a screening medium was tested on *T. indotineae* strains (n = 20) as an indication tool of TRB resistance. The obtained results were confirmed by antifungal susceptibility testing (AST) for TRB following the in vitro broth microdilution reference method. Additionally, AST was performed for eight other antifungal drugs: fluconazole, itraconazole, voriconazole, ketoconazole, griseofulvin, ciclopirox olamine, naftifine and amorolfine. Forty-five percent of the strains were confirmed to be resistant to terbinafine. The TRB resistant strains showed elevated minimal inhibitory concentration values for naftifine and amorolfine as well. DNA sequencing of the squalene epoxidase-encoding gene showed that TRB resistance was a consequence of missense point mutations in this gene, which led to amino acid substitutions F397L or L393F. MALDI-TOF MS was used as a quick, accurate identification tool for *T. indotineae*, as it can be challenging to distinguish it from closely related species such as *Trichophyton mentagrophytes* or *Trichophyton interdigitale* using morphological characteristics. While MALDI-TOF MS could reliably identify ≥ 95% of the *T. indotineae* strains (depending on the spectral library), it could not be used to successfully distinguish TRB susceptible from TRB resistant strains.

## Introduction

Dermatophytes are filamentous, keratinophilic fungi of the *Arthrodermataceae* family that are able to infect the skin, hair and nails of humans and animals. The superficial infections caused by this group of fungi are called dermatophytoses, or more frequently ringworm. Dermatophytes are generally categorized in three groups based on their primary habitat: zoophilic species are associated with animals and anthropophilic species specifically with humans, while geophilic species are associated with soil and the keratinous materials that can be found in it, such as animal hairs, feathers and horns [1, 2]. Most skin mycoses are caused by dermatophyte species belonging to the anthropophilic and zoophilic categories, and more specifically belonging to genera *Trichophyton* and *Microsporum*. Despite the superficial nature of mycoses, dermatophytes are a serious global health problem. The prevalence of skin diseases caused by dermatophytes is high: 20–25% of the global population has been estimated to suffer from these skin mycoses [3]. In addition to this common incidence, treatment of these mycoses have a considerable therapeutic cost and are a significant burden to health care systems [4, 5].

The past few years, an epidemic of skin mycoses plagued and, to this day, still affects India and its neighbouring countries. Patients mainly suffer from *tinea corporis*, *tinea cruris* or *tinea faciei*, demonstrating highly inflammatory lesions which are often difficult to treat [6, 7]. The causal agent of this Indian epidemic was labeled *Trichophyton mentagrophytes* genotype VIII after sequencing of the internal transcribed spacer (ITS) region of the ribosomal DNA. Kano et al. (2020) later reclassified the pathogen as being a separate species from *T. mentagrophytes/T. interdigitale*, naming the new dermatophyte *T. indotineae* [7–10]. Although *T. indotineae* is morphologically not distinguishable from *T. mentagrophytes*, it is transmitted from person to person and thus, anthropophilic, while *T. mentagrophytes* is a zoophilic species. *T. indotineae* also displays differences in physiology with *T. mentagrophytes* and *T. interdigitale*: the capacity to perforate hair and to hydrolyze urea is significantly lower in *T. indotineae* than in *T. mentagrophytes* and *T. interdigitale* [9]. Last but not least, the high level of resistance to the antifungal drug terbinafine is characteristic for *T. indotineae.* The elevated prevalence of terbinafine-resistant *T. indotineae* isolates can be attributed to the frequency of amino acid substitutions in squalene epoxidase at position 393 or 397 (L393F, leucine to phenylalanine; and F397L, phenylalanine to leucine), which result from point mutations in the squalene epoxidase-encoding gene (*SQLE*) [8, 11]. The observed terbinafine resistance is thought to be a consequence of excessive use of creams containing antifungal agents (such as terbinafine and azoles) and topical steroids (glucocorticoids such as clobetasol), that are sold over the counter in pharmacies, without the need for a doctor’s prescription [10].

Due to migration and globalization, terbinafine-resistant dermatophytoses caused by *T. indotineae* has been reported world-wide [10, 12–14]. In addition to its emerging prevalence over the world, it is expected that *T. indotineae* will be able to develop resistance to other antifungal drugs as well [8]. Elevated minimum inhibitory concentration (MIC) values of *T. indotineae* isolates for azoles such as itraconazole and voriconazole have already been observed [12], while cross-resistance between terbinafine and naftifine, previously described for *Trichophyton rubrum* [15], is not an unrealistic scenario. Therefore, to limit these complications and to avoid incorrect, long and fruitless treatment, it is vital to identify this pathogen as quick as possible and to determine if (terbinafine) resistance is present. Since conventional methods are often based on morphology and morphological differences between *T. indotineae, T. mentagrophytes* and *T. interdigitale* are minimal, other identifications methods, such as the accurate and quick Matrix-Assisted Laser Desorption/Ionization Time of Flight Mass Spectrometry (MALDI-TOF MS) identification tool, should be explored.

The first objective of this study was to gain more insight in the resistance profile of *T. indotineae* strains. First, a rapid screening method for TRB resistance was explored by using a selective culture medium. Secondly, antifungal susceptibility testing (AST) was performed on not only TRB, but eight other antifungal agents as well, using the in vitro broth microdilution reference method by EUCAST. These antifungal agents were fluconazole, itraconazole, voriconazole, ketoconazole, griseofulvin, ciclopirox olamine, naftifine and amorolfine. DNA sequencing of *SQLE* was used to support the obtained AST results. The second objective was to evaluate MALDI-TOF MS as a reliable identification tool for *T. indotineae*, comparing the BCCM/IHEM in-house library and the publicly accessible MSI V2.0 database.

## Material and Methods

### The Strains

Twenty *T. indotineae* strains, all originating from German patients, were isolated from the human skin between 2016 and 2019, most often causing *tinea corporis* or *tinea cruris*. The patients ages varied between 6 months and 58 years. The identity of the strains was confirmed by DNA sequencing of the ITS rDNA region (approx. 1100 bases) using primers V9G (for) and LSU 266 (rev). After sequencing, isolates were subsequently sent to the Mycology and Aerobiology unit of Sciensano (Brussels, Belgium), where they were added to the fungi culture collection of the Belgian Coordinated Collections of Microorganisms (BCCM/IHEM).

### Screening Medium

All isolates were cultured on Sabouraud dextrose agar (SDA) plates containing 0.2 μg/mL of TRB (Merck, Germany) and on a drug-free control SDA plate at 25 °C [16, 17]. Presence or absence of fungal growth was evaluated every day up until 14 days after incubation on both types of medium.

### Antifungal Susceptibility Testing

The antifungal susceptibility testing process was based on the protocol described by EUCAST for the broth microdilution antifungal susceptibility testing of filamentous fungi (E.DEF 9.4) [19]. Antifungal susceptibility testing of the *T. indotineae* strains was performed for a total of nine antifungal drugs: fluconazole, itraconazole, voriconazole, ketoconazole, terbinafine, griseofulvin, ciclopirox olamine, naftifine and amorolfine. Stock solutions of the nine antifungal drugs were prepared by dissolving the powdery drugs (Merck, Germany) in dimethyl sulfoxide (DMSO, Merck, Germany) so that the final antifungal concentrations in the microplate wells used for the inoculation of the *T. indotineae* strains ranged from 0.008 to 64 g/L. After inoculation of the *T. indotineae* strains, the microplates were incubated at 35 °C for 96 h, whereafter the MIC was visually determined at 100% inhibition of fungal growth. The microplates contained six wells with DMSO as a negative control and six without any antifungal solution as a positive control for fungal growth. Reference strains *Candida krusei* (ATCC 6258) and *Candida parapsilosis* (ATCC 22019) were used to validate the microplates. These plates were incubated at 35 °C and were read with a Biotek 96 well microplate reader (Agilent, California, USA) after 24 h of incubation.

The obtained MIC-values for the *T. indotineae* strains were compared to the wild-type upper limit (WT-UL) values established by Arendrup et al. [18, 19] and verified by EUCAST, as no epidemiological cut-off (ECOFF) values or clinical break points (CBP) have been put in place yet. For four antifungal drugs, i.e. itraconazole, voriconazole, terbinafine and amorolfine, WT-UL values were established, but this is not the case for the remaining five drugs.

### DNA Sequencing of SQLE

DNA was extracted using an eMAG® (BioMérieux, France), after incubation of a portion of the isolate for a minimum of 10 min in Lysis buffer (BioMérieux, France). *SQLE* was amplified as previously described by Moreno-Sabater et al. (2022) [18, 19]. Sequences were aligned using the sequence of *Trichophyton mentagrophytes* isolate 203,513/19 (GenBank MT700509.1). Missense mutations were screened using MEGA X (version 10.0.5).

### MALDI-TOF MS

To evaluate MALDI-TOF MS as an identification tool for *T. indotineae*, each strain was sub-cultured on Sabouraud agar amended with chloramphenicol (SC) and incubated at 25 °C for 72 h, after which protein extraction was performed following the method described by Cassagne et al*.* (2011) [20]. This method has been proven to be successful in identifying dermatophytes [21, 22]. Fungal material was gently scraped off the surface of the colonies and transferred into 300 µL of sterile water. The mixture was vortexed thoroughly and 900 µL of absolute ethanol was added subsequently. The mixture was then centrifuged for 10 min at 13 000 g, at room temperature (RT). Afterwards, the supernatant was discarded and the remaining pellet was air dried for at least 30 min, until the pellet was completely void of water. Next, the pellet was resuspended in 50 µL of 70% formic acid (Sigma-Aldrich, USA) and incubated for a minimum of five minutes at RT. The mixture was vortexed and 50 µL of acetonitrile (Sigma-Aldrich, USA) was added, again followed by an incubation period of at least five minutes at RT. The obtained suspension was centrifuged for two minutes at 13 000 g at RT. Then, 1 µL of the supernatant was pipetted onto a spot on a MALDI 96 polished steel target plate (Bruker Daltonics, Germany) and this was repeated four times (four spots per tested strain). The target plate was left to air dry at RT. After complete drying, the sample spots were covered with 1 µL of *α*-cyano-4-hydroxycinnamic acid (HCCA) matrix solution (in 50% acetonitrile, 47.5% water and 2.5% trifluoroacetic acid) and again, left to air dry at RT. Protein spectra were recorded with the Microflex LT (Bruker Daltonics, Germany) at standard settings, with a positive linear mode in a mass range from 2 to 20 kDa. The obtained spectra were analyzed with MALDI BioTyper 4.1 software (Bruker Daltonics, Germany) at standard settings using the in-house spectra library of the BCCM/IHEM Fungal Collection (Sciensano, Brussels, Belgium). The database contained six *T. indotineae* reference strains, of which three were sensitive and three were resistant to TRB. It also contained nine *T. mentagrophytes* strains and eleven *T. interdigitale* strains. The identity of all reference strains in this database was confirmed by biomolecular analysis. The MS-based identification of the tested strains was considered acceptable if the best-match log scores were ≥ 1.70 for at least three out of four spots of protein extract (with the maximum log score being 3.00).

Aside from the in-house BCCM/IHEM Fungal Collection database, the spectra were also compared to the MSI database (V2.0, accessed on 07/12/2022 in Brussels, Belgium), developed by Assistance Publique–Hôpitaux de Paris (Paris, France), Sorbonne Université (Paris, France) in collaboration with BCCM/IHEM (Sciensano, Brussels, Belgium). Since most of its reference strains were obtained after seven days of incubation and MSI results are more reliable using this incubation period, the sub-cultures of the *T. indotineae* strains were incubated for seven days instead of three days [23]. The remaining protocol steps for obtaining the protein spectra were identical to the one described above. The obtained identification scores with the MSI tool should exceed a threshold value of 20 for a reliable identification, only taking in account the spot with the highest score, with a score of 100 being the maximum score [24]. Identification can be up to species level (level of trust A) or up to genus level (level of trust B).

The ability of MALDI-TOF MS to differentiate TRB resistant from susceptible strains was evaluated as well. The.best-match reference strains were observed to investigate if MALDI-TOF MS would give reference strains with TRB resistance as a best-match when identifying TRB resistant sample strains and vice versa for susceptible strains. Using the in-house database, four spots were identified by MALDI-TOF MS per strain. The best-match reference strain was considered TRB resistant when at least 3 out of 4 spots had a TRB resistant best-match, and vice versa for TRB susceptible strains. When a strain had two spots with a TRB susceptible best-match and two spots with a TRB resistant best-match, it was noted that there was a 50/50 ratio TRB susceptible and resistant best-match strains. Using the MSI database, the reference strain with the highest score was considered the best-match and its susceptibility/resistance to TRB was taken into account. Susceptibility to TRB was unknown for some reference strains.

### Statistical Analysis

Statistical analyses were performed in order to determine significant differences in MIC values of different groups by making use of the Mann–Whitney–Wilcoxon tests. The level of significance (*p-*level) was set at 0.05. The statistical and graphic design software R was used (version 4.1.2) to execute these tests.

## Results

### Determining TRB Resistance with a Screening Medium, AST and Sequencing of *SQLE*

**Fig. 1:**
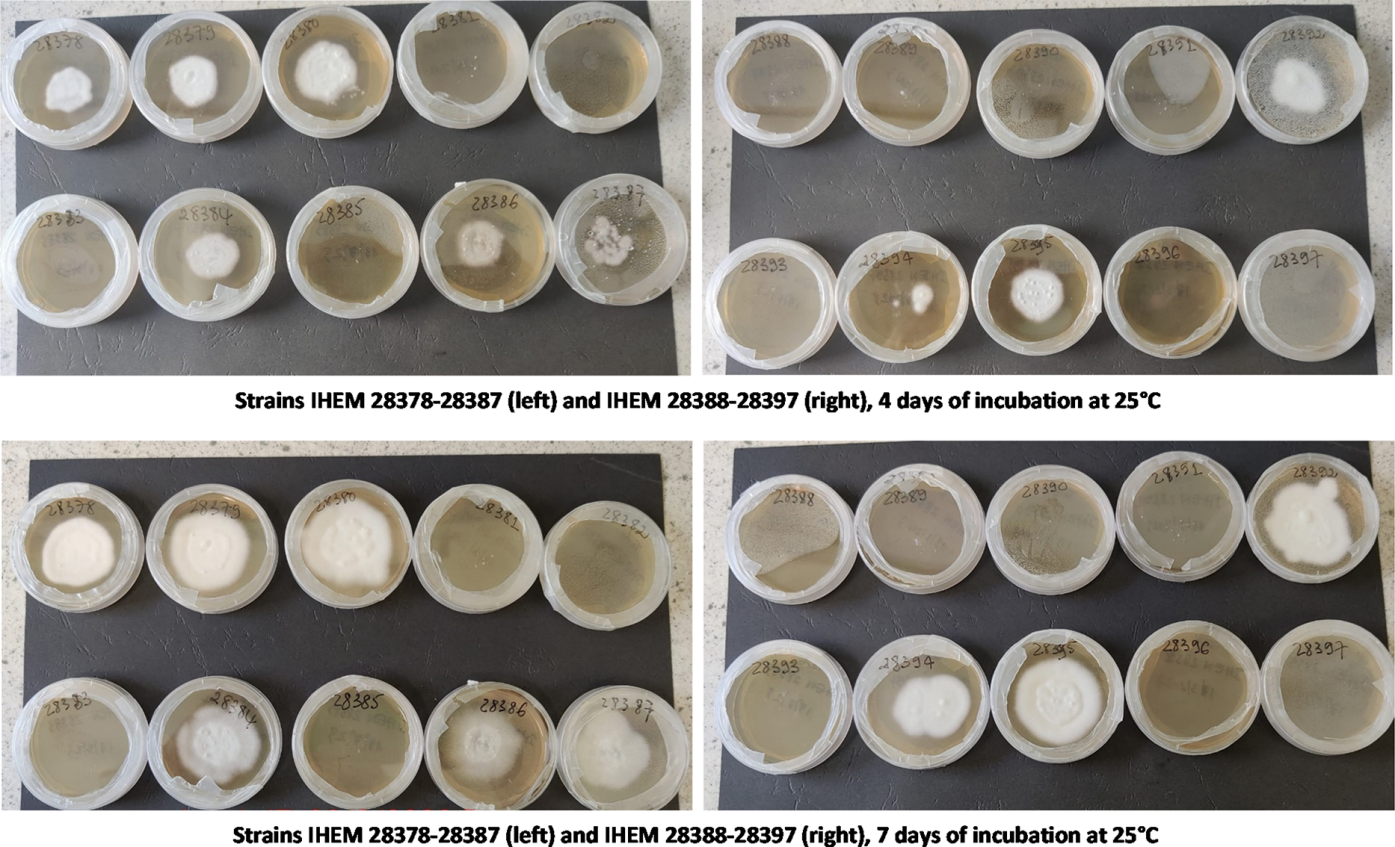
Growth of the *T. indotineae* isolates on a screening medium for terbinafine resistance. The medium consists of Sabouraud dextrose agar, amended with terbinafine. The pictures displayed in the figure were taken at four and seven days of incubation at 25 °C.

Photos of the fungal growth of all tested *T. indotineae* strains at four and seven days of incubation on the TRB containing screening medium are displayed in Fig. 1.

In Table 1, the MIC-values per *T. indotineae* strain (n = 20) can be found for the nine tested antifungal agents, obtained with the in vitro broth microdilution reference method by EUCAST. The observed *SQLE* mutations and corresponding amino acid substitutions in these strains are also displayed in this table. The overall geometric means (GM) of the obtained MIC-values are summarized in Table 2, as well as the GM for the TRB strains considered resistant or susceptible after AST.

**Table 1:** MIC-values (µg/mL) of all *T*. *indotineae *strains (n = 20) for tested antifungal agents fluconazole (FLC), itraconazole (ITC), voriconazole (VOR), ketoconazole (KET), terbinafine (TRB), griseofulvin (GRI), ciclopirox olamine (CPX), naftifine (NAF) and amorolfine (AMO). MIC-values were obtained by following EUCAST guidelines for the antifungal susceptibility testing of dermatophytes. Missense mutations in SQLE and the corresponding amino acid substitutions in the SQLE protein for the tested TRB resistant and susceptible strains. Nucleobases: thymine (T), cytosine (A), adenine (A), guanine (G). Amino acids: phenylalanine (F), leucine (L), alanine (A), threonine (T).

*T. indotineae* strains	TRB resistance	MIC (µg/mL)									*SQLE* missense mutations	Amino acid substitution
		FLC	ITC	VOR	KET	TRB	GRI	CPX	NAF	AMO		
IHEM 28378	Yes	16	0.032	0.25	0.25	16	8	1	32	0.25	T1189C	F397L
IHEM 28379	Yes	32	0.125	0.25	0.25	16	2	1	32	0.125	T1189C	F397L
IHEM 28380	Yes	64	0.25	0.25	0.5	16	4	1	32	0.125	T1189C	F397L
IHEM 28384	Yes	64	0.25	0.25	0.5	0.016	2	1	0.25	0.032	T1189C	F397L
IHEM 28386	Yes	32	0.125	0.125	0.5	0.064	2	1	0.125	0.064	C1191A	F397L
IHEM 28387	Yes	64	0.25	1	1	0.064	4	1	0.25	0.064	T1189C	F397L
IHEM 28392	Yes	16	0.125	0.25	0.25	16	4	1	32	0.125	C1191A	F397L
IHEM 28394	Yes	32	0.5	0.5	0.5	0.032	4	0.5	0.125	0.064	A1179C	L393F
IHEM 28395	Yes	32	0.125	0.25	0.25	16	4	0.5	32	0.125	T1189C & G1342A	F397L & A448T
IHEM 28381	No	64	0.25	0.5	1	16	8	0.5	32	0.25	G1342A	A448T
IHEM 28382	No	8	0.125	0.125	0.125	0.064	2	1	0.064	0.064	none	none
IHEM 28383	No	16	0.125	0.125	0.125	0.125	2	1	0.125	0.125	G1342A	A448T
IHEM 28385	No	64	0.25	0.5	0.5	0.125	4	1	0.25	0.125	G1342A	A448T
IHEM 28388	No	64	0.25	0.5	0.5	0.032	4	0.5	0.25	0.064	none	none
IHEM 28389	No	64	0.25	0.25	0.25	16	4	0.5	32	0.125	G1342A	A448T
IHEM 28390	No	16	0.064	0.125	0.125	0.064	8	0.5	0.5	0.25	G1342A	A448T
IHEM 28391	No	32	0.125	0.25	0.125	16	2	0.5	16	0.25	G1342A	A448T
IHEM 28393	No	64	0.25	0.125	0.25	16	8	0.5	32	0.25	G1342A	A448T
IHEM 28396	No	32	0.25	0.25	0.5	0.032	2	0.5	0.25	0.25	G1342A	A448T
IHEM 28397	No	16	0.064	0.125	0.125	0.032	2	0.5	0.064	0.125	G1342A & G1330A	A448T

**Table 2:** Geometric means (GM) of the MIC-values of all *T.* *indotineae* strains, and separately for the terbinafine (TRB) resistant and susceptible strains. Asterisks (*) symbolize a significant difference between the GM MIC-values of TRB susceptible and resistant strains, after statistical analysis (Mann–Whitney–Wilcoxon test, p = 0.05).

Antifungal agent	GM MIC-values (mg/L)		
	All strains (n = 20)	TRB susceptible strains (n = 11)	TRB resistant strains (n = 9)
Fluconazole	33.13	30.05	37.33
Itraconazole	0.16	0.17	0.15
Voriconazole	0.25	0.25	0.25
Ketoconazole	0.31	0.32	0.29
Terbinafine	0.67	0.05*	> 16.00*
Griseofulvin	3.48	2.92	4.32
Ciclopirox olamine	0.71	0.73	0.68
Naftifine	1.75	0.17*	29.63*
Amorolfine	0.12	0.09*	0.17*

Nine out of 20 strains (45%) were resistant to TRB (Table 1), all of them having an MIC-value of 16 mg/L or higher (as this was the upper concentration limit of the AST). These values exceed the WT-UL value established by Arendrup et al., which is set on 0.25 mg/L for terbinafine [19]. These results are in line with the observations during the screening test. At three to four days of incubation on the screening medium, growing colonies could be seen on the TRB containing medium for all TRB resistant strains, even for the slower growing strains like IHEM 28394, while none were detected at the plates incubated with susceptible strains (Fig. 1). After seven days, all TRB resistant strains were growing profusely, while the fungal growth was still zero for the susceptible strains.

For the other three antifungal agents with a WT-UL value, i.e. itraconazole, voriconazole and amorolfine (with values of 1.0, 2.0 and 1.0 mg/L respectively), all of the tested strains were considered susceptible.

When separating the TRB resistant and susceptible strains from each other and calculating the GM MIC-values for these two groups (Table 2), a clear increase in MIC-value could be spotted in resistant strains for fluconazole, griseofulvin, naftifine and amorolfine, with the difference in GMs being significant for naftifine and amorolfine (*p* = 0.05). With the exception of fluconazole, there is no noticeable difference in MIC value between TRB resistant and susceptible strains for the triazole antifungal drugs and ciclopirox olamine.

In the TRB resistant strains, the amino acid substitutions F397L (n = 8) and L393F (n = 1) could be observed, caused by *SQLE* missense mutations T1189C, C1191A or A1179C. Both substitutions F397L and L393F were absent in all 11 TRB susceptible strains. Two of the susceptible *T. indotineae* strains did not show any mutations in *SQLE*, while the remaining nine strains all contained the amino acid substitution A448T, caused by DNA mutation G134A. One susceptible strain (IHEM28397) had a silent amino acid substitution. There was one TRB resistant strain (IHEM 28395) that showed both the F397L and A448T substitutions in the SQLE protein.

### MALDI-TOF MS

#### BCCM/IHEM Database

**Fig. 2:**
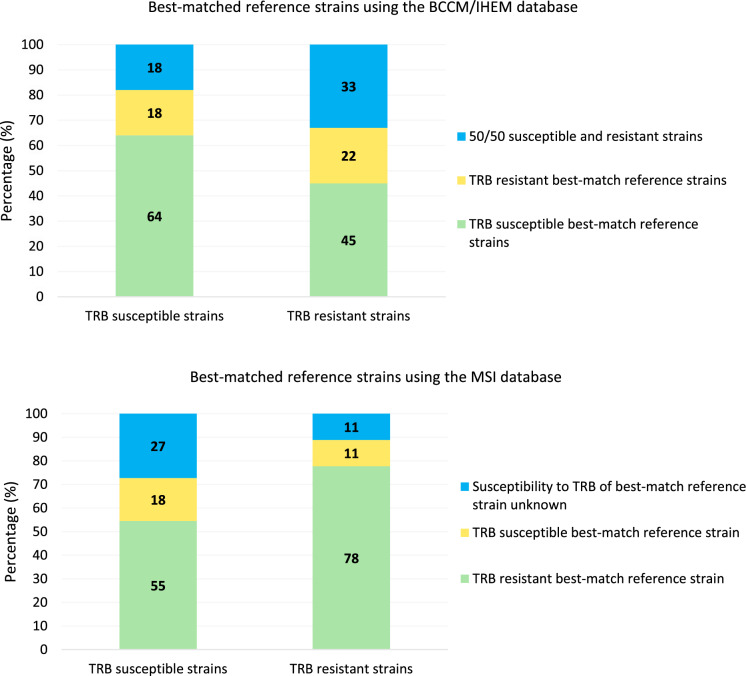
Column charts of the best-matched reference strains of the *T. indotineae* strains. **A** Best-match reference strains obtained with the in-house BCCM/IHEM library. For each strain, four spots were identified by MALDI-TOF MS, each spot linked with its best-match reference strain. The best-match reference strain was considered TRB resistant when at least 3 out of 4 spots had a TRB resistant best-match (green), and vice versa for TRB susceptible strains (yellow). When a strain had two spots with a TRB susceptible best-match and two spots with a TRB resistant best-match, a 50/50 ratio TRB susceptible and resistant best-match strains was noted (blue). **B** Best-match reference strains obtained with the MSI database. For this database, the reference strain with the highest score was taken into account (green if it was TRB resistant and yellow if it was susceptible). Susceptibility to TRB was unknown for some reference strains (blue).

After comparing the obtained spectra of the 20 tested strains with the BCCM/IHEM database, 100% of the strains was correctly identified on species-level as *T. indotineae*. An overall mean best-match log score of 2.34 ± 0.16 was observed, with mean log scores of 2.35 ± 0.16 and 2.32 ± 0.17 for TRB susceptible and resistant strains respectively (no significant difference). The first three given best-match reference strains were *T. indotineae* strains for each tested strain. At the mass range and settings of the MALDI-TOF MS instrument, no distinction between TRB susceptible and resistant strains could be made by analyzing the best matches (Fig. 2).

#### MSI V2.0 Database

Using the MSI platform, 95% of the tested *T. indotineae* strains were correctly and reliably identified on species-level (index of trust A). While the remaining 5% was also correctly identified as *T. indotineae*, the identification was only considered reliable on genus-level (index of trust B). The mean score of all strains was 58.68 ± 5.75, with 58.29 ± 6.04 and 58.99 ± 5.77 for TRB resistant and susceptible strains. Similar to the BCCM/IHEM database, a differentiation between TRB resistant and sensitive strains could not be made by MALDI-TOF MS (Fig. 2).

## Discussion

A screening medium could be a useful tool in the determination of possible TRB resistance. This method can be seen as a fast first screening step before performing thorough antifungal susceptibility testing. The EUCAST AST protocol can be expensive and time-consuming: for dermatophytes like *T. indotineae*, a growing period of five to seven days before inoculation and an incubation period of two to five days after inoculation are required [25]. Using this screening medium, resistance to TRB could be detected within four days, while performing AST takes minimum a week, in all probability even longer. Nevertheless, in order to have a complete overview on susceptibility profiles and the evolution of TRB resistance levels, thorough AST using the EUCAST standardized method remains primordial.

When performing EUCAST AST on the twenty *T. indotineae* strains, nine strains (45%) were considered resistant to terbinafine. Eight of them contained the amino acid substitution F397, of which earlier research has shown that it is known to contribute to TRB resistance in *T. indotineae* [13, 26, 27]. In one of the resistant strains, amino acid substitution L393F was spotted, which has also already been observed in TRB resistant *T. indotineae* strains [12, 13, 26].

Aside from terbinafine, the TRB resistant strains showed increased MIC-values for naftifine as well. Naftifine is, like terbinafine, an allylamine which inhibits squalene epoxidase and thus, ergosterol production, which is a necessary component for fungal cell membranes. As naftifine exhibits the same mode of action as terbinafine, cross-resistance of these two antifungal agents is suspected. This phenomenon has been observed previously by Mukherjee et al. [15] for *T. rubrum*, who also described cross-resistance with other squalene epoxidase inhibitors such as butenafine and tolnaftate. In research by Rudramurthy et al. [28], the F397L substitution caused elevated MIC-values for naftifine in *T. rubrum* strains, also causing cross-resistance to TRB.

*While T. indotineae* TRB resistant strains were not declared resistant to amorolfine when considering the WT-UL values established by EUCAST, a significant increase in MIC-value could be observed in comparison with TRB susceptible strains. Amorolfine is an morpholine antifungal drug, inhibiting ergosterol production by interfering with Δ^14^-sterol reductase and Δ^8→7^ -isomerase [29], which is a different mode of action than TRB’s. Nevertheless, resistance to both naftifine and TRB has been described in *C. glabrata* [30, 31], although the resistance has been linked to overexpression of ATP-binding cassette (ABC) transporter genes instead of *SQLE* mutations. Fungal ABC transporters can act as efflux pumps for several antifungal drugs, providing resistance of the fungus [32, 33]. Ghelardi et al. [31] made notion of cross-resistance of amorolfine resistant *T. rubrum* strains to terbinafine, also mentioning overexpression of ABC transporters as the most probable explanation. Aside from ABC transporter overexpression, the observed *SQLE* mutations could not only have impacted squalene epoxidase, but other steps further in the ergosterol synthesis as well, leading to an increase in MIC-values.

There was no significant increase in MIC-values for all azole antifungal drugs when comparing TRB resistant strains to susceptible ones, although elevated values could be observed for fluconazole. Nine of the eleven TRB susceptible strains contained the A448T amino acid substitution. This substitution is known to cause increased MIC-values for triazole antifungal drugs, but there were no significantly elevated values observed in this research. These results are in line with the findings of Kong et al. [27]. They showed that the A448T substitution does not cause higher MIC-values for triazoles on its own, but it can lead to TRB resistance when present together with substitution F397L.

MALDI-TOF MS is known for its accurate and fast method for identifications of fungal species, which makes it a frequently used alternative in laboratories for identification by morphology or molecular sequencing. Morphology-based identification is still considered the conventional method for identification of fungi, but it can be time-consuming. This is especially the case for dermatophytes, because the fungus often needs to be cultured and grown for a sufficient amount of time before distinctive morphological characteristics can be seen under the microscope. Moreover, highly skilled staff is necessary to distinguish the features that are characteristic for each species [21]. This is especially the case for closely related species such as *T. indotineae,* which is phenotypically similar to *T. mentagrophytes*/*T. interdigitale.* Recently, Normand et al. [23] highlighted the capacity of the online MSI-2 application to correctly identify *T. indotineae* using MALDI-TOF MS. In this study, we evaluated the BCCM/IHEM in-house library for identification of twenty *T. indotineae* strains. Each isolate was correctly identified on species-level, while the library contained several reference spectra of *T. mentagrophytes* and *T. interdigitale* as well. When a self-managed in-house database is not available or does not give the desired accuracy of identification, the online and free MSI V2.0 tool can be used. Using this platform, 100% of the strains were correctly identified as *T. indotineae*, of which 95% were considered reliable on the species-level (level of trust A).

When analyzing the reference spectra concurring with the highest identification scores for the tested *T. indotineae* strains, no relation could be found between reference spectra of TRB resistant strains and spectra of resistant test strains. The same was true for TRB susceptible strains. This means that even if the mean of the best-match log score of a *T. indotineae* strain originates from a resistant strain, no confirmation can be given that the tested strain is resistant as well. This could possibly be due to the lack of range of analysis of the Microflex LT, since the observed TRB resistance is often the consequence of a single mutation in *SQLE.* In the future, with advanced specificity or detection, spotting resistance could be possible by using the MALDI-TOF MS technique. In this way, identification and resistance screening could be performed at the same time, using only one lab technique. There are several studies testing the capability of MALDI-TOF MS to detect resistance to antifungal drugs in fungi. In some researches, comparison between mass spectra of fungi incubated on different concentrations of antifungal drugs is used to obtain a composite correlation index, of which its value indicates if antifungal resistance is present or not [34–36]. A second technique is antifungal susceptibility testing by the means of the MALDI Biotyper antibiotic susceptibility test rapid assay (MBT ASTRA), which is a phenotypic assay comparing fungal growth after incubation on different concentrations of antifungal drugs by using the area under the curve of the obtained mass spectra [36, 37]. Especially the latter technique has shown some promising results in some *Candida* species.

## Conclusion

In conclusion, this research demonstrated that a screening medium consisting of Sabouraud agar containing TRB could screen TRB resistance in *T. indotineae* strains in four days, which can be considered as a rapid first screening before performing broth microdilution antifungal susceptibility testing or when such type of analysis is not possible. After antifungal susceptibility testing, significantly elevated MIC-values were observed in TRB resistant *T. indotineae* strains for naftifine and amorolfine, raising the possibility of cross-resistance. The observed TRB resistance was caused by point mutations in *SQLE*, mainly resulting in amino acid substitutions F397L and L393F, while amino acid substitution A448T does not seem to cause azole resistance in *T. indotineae* when no other substitutions are present. Additionally, this study demonstrated the strength of MALDI-TOF MS as a reliable tool for the identification of *T. indotineae* strains, as it is able to distinguish them from closely related species *T. interdigitale* and *T. mentagrophytes*. Nevertheless, a distinction between TRB resistant and susceptible strains could not be made by this spectrometric technology.
